# Association Between A-Waves and Outcome in Pediatric Guillain-Barré Syndrome

**DOI:** 10.3389/fneur.2022.914048

**Published:** 2022-06-17

**Authors:** Mei Jin, Jing Liu, Ziwei Zhao, Wenjin Geng, Suzhen Sun

**Affiliations:** Department of Pediatric Neurology, Children's Hospital of Hebei Province, Shijiazhuang, China

**Keywords:** Guillain-Barré syndrome, children, A-waves, Hughes grade, outcome

## Abstract

**Introduction:**

To examine the importance of abundant A-waves in electrophysiological classification and prognosis of pediatric Guillain-Barré Syndrome (GBS).

**Methods:**

A single-center and retrospective study enrolling 65 children-patients, aged 16 years and younger, with clinically diagnosed GBS between 2013 to 2020. Hughes grade was used to assess functional disability at nadir, 1 month, and 6 months after symptom onset. Patients were divided into 2 groups according to the presence of abundant A-waves. Clinical features and prognosis between the 2 groups were compared.

**Results:**

The distal motor latency of the median nerve in patients with GBS with A-waves (9.18 ms) was more prolonged than that of patients with GBS without A-waves (4.1 ms). An electrophysiological variant of these two groups was also statistically different (*p* = 0.006). The short-term prognosis of patients with AIDP with A-waves was worse than patients with AIDP without A-waves (χ^2^ = 5.022, *p* = 0.025), and univariable logistic regression analysis showed statistically significant (OR: 5.844, 95% CI 1.118–30.553; *p* = 0.036).

**Conclusion:**

A-waves were strongly associated with demyelination and poor short-term prognosis of AIDP in children. We proposed an electrophysiological marker for early prediction of outcome in the AIDP subtype of GBS, applicable for clinical practice and future treatment administration.

## Introduction

Guillain-Barré syndrome (GBS) is an acute polyradiculoneuropathy characterized by progressive limb weakness with or without paresthesia (1). Most children with GBS have good prognoses, however, some patients were unable to walk unaided within the first 6 months. Therefore, early identification of risk factors for poor prognosis is critical for patients with GBS to prevent irreversible nerve degeneration. A-waves are late responses recognized during the recording of F waves (2). Many studies of adult patients with GBS have shown that A-waves, as a novel marker of demyelination, carried poor prognostic value (3, 4). Few studies have examined the association between A-waves and clinical features, as well as outcomes in children with Guillain-Barré syndrome. We aimed to identify the clinical importance of A-waves in electrophysiological classification and prognosis of pediatric Guillain-Barré syndrome.

## Materials and Methods

### Subjects

We retrospectively recruited patients (aged 16 years and younger) admitted to our Neurology Units between 2013 and 2020. Patients met level 2 of the Brighton classification of GBS (5, 6). Clinical and Nerve Conduction Study (NCS) data were collected within 4 weeks of symptom onset. Patients with Miller Fisher syndrome and other causes of neuropathies, such as acute transverse myelitis, and chronic inflammatory demyelinating polyradiculoneuropathy, were excluded. This study was approved by the Ethics Committee of the Children's Hospital of Hebei Province.

### Methods

#### NCS Methods

The NCS studies were recorded using an electromyogram evoked potential system MEB2306C (Japan). Motor nerve conduction studies were performed on median, ulnar, tibial, and peroneal nerves. F-waves were examined at median, ulnar, and tibial nerves. Sensory nerve conduction studies were performed on median, ulnar, and sural nerves. All patients received full NCS studies twice within 4 weeks after symptom onset and were classified using the criteria of Ho et al. (7) into acute inflammatory demyelinating polyneuropathy (AIDP), acute motor axonal neuropathy (AMAN), or unclassified. A-waves were analyzed by recording F-wave. A-waves of the median or ulnar nerves were mainly considered in the present study because a few A-waves may be observed even in the tibial nerve of normal subjects. Three or more A-wave peaks were termed “abundant A-waves”. The patients were divided into two groups according to the presence or absence of abundant A-waves.

#### Motor Functional Disability Assessment

The patient's motor functional disability was assessed by the Hughes grade (8) at nadir, 1 month, and 6 months after symptom onset. Patients with a Hughes grade of ≥3 at 1 month were considered poor outcomes, whereas patients with a Hughes grade of <3 were considered good outcomes.

#### Clinical and Laboratory Data

The clinical features of bulbar paralysis, facial paralysis, autonomic dysfunction, and mechanical ventilation were analyzed. Laboratory findings, such as the protein levels of cerebrospinal fluid (CSF), anti-glycolipid antibodies of serum and CSF, and T/B lymphocyte of serum were also measured.

#### Statistical Analyses

Categorical data of clinical and electrophysiological features were shown as proportions and compared using a chi-square test or Fisher's exact probability test. Continuous data of clinical and electrophysiological features were shown as the medians with IQR and tested by the Wilcoxon rank-sum test. A-wave as a potential prognostic factor at 1months after symptom onset was analyzed by univariable logistic regression analysis. A *p-*value of 0.05 was significant.

## Results

### Baseline Clinical Features

A total of 65 patients (38 male, 27 female) were recruited, and patients with GBS with or without A-waves were 32 (49.2%) and 33 (50.8%) cases, respectively. Cerebrospinal fluid (CSF) protein levels of patients with GBS with A-waves (1g/L) were significantly higher than patients with GBS without A-waves (0.76g/L). Meanwhile, the distal motor latency of the median nerve in the patients with GBS with A-waves (9.18 ms) was more prolonged than that of patients with GBS without A-waves (4.1 ms). In addition, an electrophysiological variant of these two groups was also statistically different (*p* = 0.006), which was abundant A-waves that mainly occurred in the AIDP subtype and did not occur in the AMAN subtype. Other clinical features and electrophysiological data of pediatric GBS were summarized in Table 1.

**Table 1:** Clinical and electrophysiological data in pediatric Guillain-Barré syndrome (GBS).

**Variables**	**Patients with GBS with A-waves** **(*n =* 32)**	**Patients with GBS without A-waves** **(*n =* 33)**	**Statistic values**	***P-value***
Age, years, median (IQR)	5 (3–6)	6 (3–9)	*Z* = 1.19	0.234
Male, *n* (%)	21 (65.6)	17 (51.5)	χ^2^ = 1.332	0.248
Preceding event, *n* (%)				0.609^a^
Respiratory infection	23 (71.9)	21 (63.6)		
Gastrointestinal infection	1 (3.1)	2 (6.1)		
From onset to admission, days, median (IQR)	5.5 (3–10)	6 (3–9)	*Z* = 0.416	0.678
From onset to nadir, days, median (IQR)	5.5 (4–10)	6 (3–9)	*Z* = 0.304	0.761
Hughes scores at nadir, grade, median (IQR)	4 (3–4)	4 (3–4)	*Z* = 0.821	0.412
**Neurological symptoms**, ***n*** **(%)**				
Facial paralysis	6 (18.8)	2 (6.1)	—	0.149^a^
Bulbar paralysis	10 (31.3)	11 (33.3)	χ^2^ = 0.032	0.857
Neuropathic pain	19 (59.4)	17 (51.5)	χ^2^ = 0.406	0.524
Autonomic dysfunction, *n* (%)	10 (31.3)	17 (51.5)	χ^2^ = 2.747	0.097
Mechanical ventilation, *n* (%)	2 (6.3)	7 (21.2)	—	0.149^a^
Distal motor latency of median, ms, median (IQR)	9.18 (4.9–10.88)	4.1 (2.85–7.7)	*Z* = 2.565	0.01
Variant, *n* (%)			—	0.006^a^
AIDP	27 (84.4)	19 (57.6)		
AMAN	0	8 (24.2)		
Unclassified	5 (15.6)	6 (18.2)		
Proteins in CSF, g/L, median (IQR)	1 (0.79–1.26)	0.76 (0.48–1.03)	*Z* = 2.459	0.014
T/B lymphocyte abnormalities, *n* (%)	24 (92.3)	21 (87.5)	—	0.661^a^
Anti-glycolipid antibody positive, *n* (%)	3 (12.5)	4 (18.2)	—	0.694^a^
**Treatment**, ***n*** **(%)**				
IVIg	32 (100)	33 (100)	NA	
Plasmapheresis	1 (3.1)	4 (12.1)	—	0.355^a^
Hughes score at 1 month after onset, *n* (%)			—	0.038^a^
0	3 (9.4)	7 (21.2)		
1	11 (34.4)	8 (24.3)		
2	5 (15.6)	12 (36.4)		
3	9 (28.1)	2 (6.1)		
4	3 (9.4)	1 (3)		
5	1 (3.1)	3 (9)		
Hughes score at 6 months after onset, *n* (%)			—	0.262^a^
0	22 (68.8)	26 (78.8)		
1	4 (3.1)	5 (15.2)		
2	1 (12.5)	2 (6.1)		
3	3 (9.4)	0		
4	2 (6.3)	0		
Duration of hospitalization, days, median (IQR)	19 (13–27.3)	19 (14–29)	Z = 0.723	0.470

*GBS, Guillain-Barré syndrome; AIDP, acute inflammatory demyelinating polyneuropathy; AMAN, acute motor axonal neuropathy*.

a *Fisher's exact test. Z, Rank sum test; NA, not applicable*.

### Association of A-Waves and Clinical Severity and Outcome

Hughes grades were no statistical differences at nadir and 6 months after symptom onset both between patients with GBS with A-waves vs. without A-waves and between patients with AIDP with A-waves vs. without A-waves (all *p* > 0.05). Hughes scores of patients with GBS with A-waves at 1 month were worse than those of patients with GBS without A-waves (Fisher's exact test, *p* = 0.038) (Table 1), however, univariable logistic regression analysis of the potential predictive value of the A-waves concerning unable to walk unaided (Hughes grade of 3 and more) within 1 month showed no statistical difference (OR: 3.079, 95% CI 0.993–9.545; *p* = 0.051) (Table 2). Hughes scores of patients with AIDP with A-waves at 1 month were worse than those of patients with AIDP without A-waves (*p* = 0.043) (Table 3). Meanwhile, univariable logistic regression analysis showed statistical difference (OR: 5.844, 95% CI 1.118–30.553; *p* = 0.036).

**Table 2:** A-wave as a risk of poor outcome, defined as the inability to walk unaided at 1 month since symptom onset based on binary logistic regression analysis.

	**No**.	**OR (95% CI)**	***p***
**Patients with GBS**			
with A-waves	32	3.079 (0.993–9.545)	0.051
without A-waves	33	1.00	
**Patients with AIDP**			
with A-waves	27	5.844 (1.118–30.553)	0.036
without A-waves	19	1.00	

*GBS, Guillain-Barré syndrome; AIDP, acute inflammatory demyelinating polyneuropathy*.

**Table 3:** Clinical features and Hughes grade between patients with AIDP with A-waves and patients with AIDP without A-waves.

**Variables**	**Patients with AIDP with A-waves** **(*n =* 27)**	**Patients with AIDP without A-waves** **(*n =* 19)**	**Statistic values**	***P-value***
Age, years, median (IQR)	11 (4–11)	6 (1–6)	*Z* = 0.427	0.669
Male, *n* (%)	19 (70.4)	9 (47.4)	χ^2^ = 2.477	0.116
Hughes scores at nadir, grade, median (IQR)	5 (4–5)	4 (3–4)	*Z* = 0.059	0.953
Hughes score at 1 month after onset, *n* (%)			—	0.043^a^
0	2 (7.4)	5 (26.3)		
1	10 (37)	5 (26.3)		
2	4 (14.8)	7 (36.8)		
3	9 (33.4)	1 (5.3)		
4	1 (3.7)	1 (5.3)		
5	1 (3.7)	0		
Hughes score at 6 months after onset, *n* (%)			—	0.098^a^
0	19 (70.4)	19 (100)		
1	4 (14.8)	0		
2	1 (3.7)	0		
3	1 (3.7)	0		
4	2 (7.4)	0		

*AIDP, acute inflammatory demyelinating polyneuropathy*.

a *Fisher's exact test*.

## Discussion

Guillain-Barré syndrome is currently the most common cause of acute flaccid paralysis in children. The prognosis of patients with GBS is considered good; however, approximately 20% of patients are unable to walk unaided (9), and about 7% of patients died (10). As a special group, most children with GBS have a good prognosis (11, 12). In this study, 29.2% (19/65) of patients at 1 month and 7.7% (5/65) of patients at 6 months had poor prognoses, respectively. Therefore, early identification of risk factors for poor prognosis is critical in patients with GBS, who are eligible for additional effective treatment to reduce the occurrence of adverse events and prevent irreversible nerve degeneration.

Many studies of adult patients with GBS have shown that the electrophysiological technique plays an important role in early diagnosis and subtype classification, and is correlated with prognosis (13–15). A-waves are usually defined as indirect components under supramaximal stimulations, just like an F-wave (16, 17), the possible mechanisms of A-waves are proximal re-excitation, and not because of axon reflex and unidirectional ephaptic transmission (18, 19). In the present study, abundant A-waves were associated with prolonged distal motor latency and mainly occurred in the AIDP subtype (84.4%) and did not occur in the AMAN subtype, therefore, abundant A-waves play an important role in the early diagnosis of demyelination subtype of Guillain-Barré syndrome.

A close correlation between abundant A-waves and Guillain-Barré Syndrome has been suggested by adult patients with GBS (18, 20), however, they have been rarely studied in pediatric Guillain-Barré syndrome. In this study, there was a weak correlation between abundant A-waves and the poor short-term prognosis of patients with GBS. It could be explained that the outcome of the axonal subtype was worse than that of the demyelination subtype, and all AMAN subtypes were categorized into patients with GBS without A-waves, therefore, the axonal subtype may have a possible effect on the prognosis of patients with GBS. To better explore the A- waves value in the prognosis of the AIDP subtype and avoid the possible axonal subtype effect, univariable logistic regression showed potential predictive value of the A-waves, revealing that abundant A-waves were strongly correlated with poor short-term prognosis of patients with AIDP, and it could also alert physicians in the early stages of the disease to take active and effective combination therapy to reduce the poor prognosis and shorten the course of the disease.

In addition to electrophysiological study, albuminocytological dissociation and anti-glycolipid antibody of CSF could further support the diagnosis of GBS (21, 22). In this study, the patients with GBS with A-waves had a higher protein level than patients with GBS without A-waves (*p* < 0.05). This phenomenon pathologically showed that patients with A-waves had more severe demyelination and axonal damage, and therefore, had a poor prognosis.

Our study was also subject to some limitations. Firstly, this study did not analyze the correlation between A-wave and compound muscle action potential, future efforts should focus on the combined electrophysiological model to predict the clinical severity and outcome for patients with GBS. Secondly, one or two peaks of A-waves were not considered in the present study, because 3 or more peaks of A-waves were the optimal cut-off value to discriminate between AIDP and AMAN subtypes, the recognition and counting of A-waves peaks may be a somewhat arbitrary task. Moreover, this was a retrospective study, we will further conduct some prospective clinical studies on the prognosis of GBS in children based on the present study.

In conclusion, abundant A-waves, as a reliable marker of demyelination, play an important role in the early diagnosis of the AIDP subtype and were strongly associated with poor short-term prognosis of AIDP in children. We proposed an electrophysiological marker for early prediction of outcome in the AIDP subtype of GBS, applicable for clinical practice and future treatment administration.

## Data Availability Statement

The original contributions presented in the study are included in the article/supplementary material, further inquiries can be directed to the corresponding author.

## Ethics Statement

The study was approved by the Ethics Committee of Children's Hospital of Hebei Province with written informed consent from all subjects. All subjects provided written informed consent in accordance with the Declaration of Helsinki. Written informed consent to participate in this study was provided by the participants' legal guardian/next of kin.

## Author Contributions

JL acquired the electrophysiological data. ZZ and WG collected serum, CSF samples, and completed the statistical analysis. MJ designed the experiments, interpreted the results, and drafted the initial manuscript. SS revised the initial draft and wrote the final manuscript. All authors contributed to the article and approved the submitted version.

## Funding

This work was supported by grants from the Medical Science Research Key Project Plan of Hebei Province in 2020 (20200223).

## Conflict of Interest

The authors declare that the research was conducted in the absence of any commercial or financial relationships that could be construed as a potential conflict of interest.

## Publisher's Note

All claims expressed in this article are solely those of the authors and do not necessarily represent those of their affiliated organizations, or those of the publisher, the editors and the reviewers. Any product that may be evaluated in this article, or claim that may be made by its manufacturer, is not guaranteed or endorsed by the publisher.
